# Fungal communities in Florida salt marsh mosquito midguts vary between species and over time but have low structure

**DOI:** 10.3389/fimmu.2025.1648091

**Published:** 2025-09-23

**Authors:** Daniel W. Pérez-Ramos, Eric P. Caragata

**Affiliations:** Florida Medical Entomology Laboratory, Department of Entomology & Nematology, Institute of Food & Agricultural Sciences, University of Florida, Vero Beach, FL, United States

**Keywords:** mosquito, microbiome, mycobiome, fungi, microbial ecology, rhodotorula

## Abstract

**Introduction:**

Microorganisms are intrinsically tied to the developmental and reproductive success of mosquitoes, can influence their ability to resist insecticides, and can strongly influence their ability to harbor and transmit pathogens of medical importance. Although mosquito-associated fungi have oben been overlooked at the expense of bacteria, several different fungal taxa are known to modulate interactions between mosquitoes and pathogens, while others have potential applications as biopesticides due to their entomopathogenic activity. Accordingly, understanding how and why different fungi associate with mosquito tissues is an important step toward elucidaUng the impact the diverse kingdom of microorganisms has on mosquito biology and mosquito- borne disease.

**Methods:**

In this study, we used Illumina Mi-Seq profiling of the internal transcribed spacer gene to characterize the midgut mycobiota of field collected adult mosquitoes from three species: Aedes taeniorhynchus, Anopheles atropos, and Culex nigripalpus, at two different collection times.

**Results:**

We observed that all mosquito specimens carried high loads of Rhodotorula lamellibrachiae, a common environmental yeast that is known to be involved in nitrogen fixation, although its role in mosquito biology is not clear. We also find that the mycobiome is strongly influenced by mosquito species, that few fungi have both high abundance and prevalence, and that few fungi consistently co- associate across time and host species.

**Discussion:**

These findings suggest that there is limited structure to mosquito associated fungal communities, implying that their assembly may be more driven by stochastic than deterministic processes. Our findings highlight the influence of key variables on mosquito fungal diversity and help facilitate understanding of how and when mosquitoes acquire fungi and the roles that fungi play in mosquito biology.

## Introduction

Vector-borne diseases are caused by pathogens spread by arthropods, including mosquitoes. These diseases represent a major threat to global public health, with more than half of the world’s population at risk, particularly in tropical and subtropical regions. Cases of dengue have increased drastically over the past two decades, with an estimated 100–390 million infections occurring every year (1). An estimated 250 million cases of malaria occur annually (2), with recent resurgences (3) offsetting declines that had been observed since the turn of the century (4). Cases of encephalitic viruses, including Japanese encephalitis virus, St. Louis encephalitis virus (SLEV), and West Nile virus (WNV), remain an ongoing threat in many parts of the world (5). In the absence of medical treatments or the availability of vaccines to the general public, the most common method of disease management is through mosquito population abatement. However, many long-standing mosquito control programs have become hampered by the emergence of resistance to commonly used insecticidal chemicals, which is now highly prevalent amongst mosquito populations (6–8). Accordingly, there has been a push to develop novel mosquito abatement strategies.

Control of mosquito populations might be achieved through manipulation of the microbiota, the community of bacteria, fungi, viruses, and protozoan parasites that naturally associate with mosquito tissues (9, 10). The mosquito microbiota is diverse, with high levels of variation in microbial community composition observed between individual mosquitoes, mosquito populations (11), mosquito species (12), and due to extrinsic variables such as collection time, seasonality, land cover, and collection location (13–16). However, the influence of these variables on the microbiome is not uniform across studies, which suggests that a complex series of factors combine to determine which microorganisms come to associate with a given mosquito population at a given time. This is important because the presence or absence of the microbiota and the presence of specific microbial taxa can modulate many mosquito physiological processes, including juvenile development (17), oviposition behavior (18, 19), blood feeding and egg production (20), immunity (21, 22), longevity (23), and the ability to resist insecticides (24). In this framework, extrinsic factors help to determine which microorganisms associate with mosquitoes, resulting in variation in the composition of the microbiome, which has the potential to produce different fitness outcomes for mosquitoes and potentially impact population-level parameters such as population size, age structure, and vectorial capacity.

Furthermore, there is a growing body of evidence that suggests that the microbiome can modulate a mosquito’s ability to harbor and transmit pathogens of medical importance (21, 22). One of the most well-known examples is *Wolbachia pipientis*, an intracellular bacterium that can block arboviral infections in mosquitoes (25) but also induces a natural form of genetic drive known as cytoplasmic incompatibility (26). These two properties have allowed *Wolbachia* to be used in large-scale interventions that seek to suppress *Aedes* mosquito populations (27) or render mosquito populations less permissive to a target pathogen (28). Anti-pathogen effects have also been observed in other bacteria, including *Chromobacterium* Csp_P, which produces an enzyme that directly lyses the dengue envelope protein (29); *Rosenbergiella*_YN46, which acidifies the mosquito gut and inactivates viral particles (30); and *Asaia* sp., which induces an immune response in *Anopheles* mosquitoes resulting in reduced *Plasmodium berghei* load (31). However, there are also examples of microorganisms that have pro-pathogen activity, including *Serratia odorifera*, which produces a polypeptide that enhances viral infection (32), and *Serratia marcescens*, which produces a protein that promotes viral invasion of the mosquito midgut epithelial cells (33). These pro- and anti-pathogen effects have not been examined for the vast majority of mosquito-associated microorganisms.

Although most studies of the mosquito microbiome are concerned with the bacterial microbiota, other types of microorganisms also play important roles in mosquito biology, including mosquito immunology. Fungal infection in mosquitoes prompts a strong melanization response and humoral immunity (34) as well as different innate immune signaling cascades (35, 36) via the Toll (37), IMD (38), or JAK/STAT (38, 39) pathways, which result in the production of antimicrobial peptides. An initial fungal infection in mosquitoes can also impact the subsequent immunological state of mosquitoes. For instance, infection with the entomopathogenic fungus *Beauveria bassiana* in *Ae. aegypti* mosquitoes prompt the expression of thioester-containing immune proteins, augmenting susceptibility to further fungal infections (38). Meanwhile, the presence of the commensal fungus *Penicillium chrysogenum* induces ornithine decarboxylase expression, which sequestrates L-arginine and enhances *Plasmodium falciparum* infection rates in *An. gambiae* mosquitoes (40).

Both mosquito-associated fungi and viruses can modulate mosquito-pathogen interactions. The presence of two widespread insect-specific viruses in *Aedes* spp. mosquitoes increase the likelihood that they can transmit the Zika and dengue viruses to new vertebrate hosts (41). The presence of a *Talaromyces* sp. fungus makes *Aedes aegypti* more susceptible to DENV infection (40), while the presence of a *Penicillium* sp. fungus increase susceptibility to *Plasmodium* infection in *Anopheles gambiae* (42). Interestingly, pathogen infection can alter the diversity of mosquito-associated fungi, as is the case with *Aedes triseriatus* and *Aedes japonicus*, where fungal richness decreased after La Crosse virus infection (43), suggesting that there is cross-reactivity with the mosquito immune system.

Fungi can also be of interest to mosquito abatement because of their natural entomopathogenic activity against mosquitoes, with specimens from several taxa displaying mosquitocidal activity (44–46). Several of these fungi have been explored as novel biopesticides, and some have been utilized to enhance the performance of bed nets intended to prevent night biting by mosquitoes (47, 48). However, the mosquito mycobiome is made up of many other taxa that do not necessarily have negative impacts on mosquito survival, including both filamentous fungi and yeasts (49, 50). The functional niches filled by the majority of these organisms are uncharacterized. Like bacteria (51), many fungi appear to be acquired from the environment by mosquito larvae and then persist in mosquito adults (52, 53). In *Aedes albopictus*, fungal diversity varies between populations of the same species (54), and both the midgut and the crop harbor a high number of fungal taxa (55). It is unclear whether environmental heterogeneities influence the diversity and composition of the mycobiota as they do with the bacterial microbiota.

To build knowledge of mosquito-associated fungal communities, we sought to assess the fungal diversity of natural mosquito populations in a mangrove swamp, which, in our previous study (56), demonstrated high levels of bacterial diversity in mosquitoes. We examined three mosquito species of medical or veterinary relevance. *Aedes taeniorhynchus* is a salt marsh mosquito and a suspected vector of several arboviruses and dog heartworm *Dirofilaria immitis* (57) that is found in the eastern United States from Massachusetts to Florida and in southern coastal Texas (58). *Anopheles atropos* is a suspected vector of WNV (59), which is found on the east coast from Florida up to New Jersey and in southern, coastal Texas (58). *Culex nigripalpus* is a known vector of SLEV, WNV, and *D. immitis* (60), which has a broader distribution, covering many states in the southern and southeastern United States, as well as more central states including Kentucky and Tennessee (58).

We wanted to examine the influence of mosquito species and the time of the year when mosquito specimens were collected on mycobiome composition and diversity. To do so, we collected mosquito specimens using CO_2_-baited mini light traps and selected specimens from the three mosquito species for Illumina Mi-Seq sequencing using the Internal Transcribed Spacer (ITS) 1–2 region. Through fungal community ecology analyses, we demonstrate that despite a relative lack of fungal community structure, both collection time and mosquito species strongly influence the fungi found in the mosquito midgut, but mosquito species had the stronger influence. We also reveal the ubiquity of yeasts from sub-division Basidiomycota across all mosquito specimens, with these putative nitrogen-fixing microorganisms well placed to play an important role in mosquito biology due to their high prevalence and abundance. Critically, our findings suggest that the nature of fungal communities in natural mosquito populations and the influence of environmental factors on these communities appear to differ from what occurs with the bacterial microbiota, suggesting that ecological factors that drive their assembly and their role in mosquito biology may differ.

## Materials and methods

### Study site

**Figure 1:**
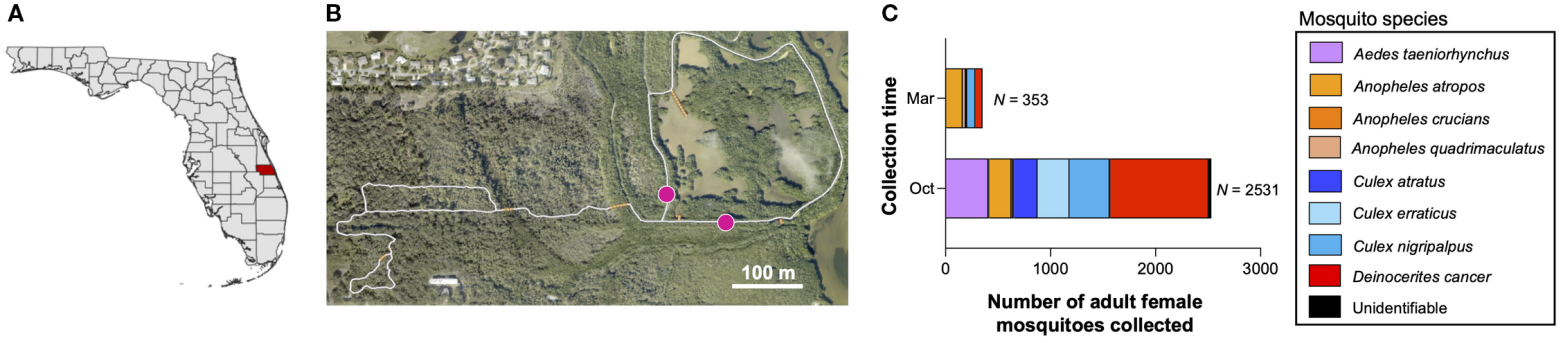
Mosquito collection details. Mosquito specimens in this study were collected from the Oslo Riverfront Conservation Area (ORCA), Vero Beach, Indian River County (marked in red), FL, USA, which is located in the central east coast of the Florida peninsula **(A)**. A map highlighting the study trap locations set out approximately 100 m from each other in the ORCA mangroves wetlands. One dry ice-baited CDC mini light trap was deployed at each location marked on the map (magenta circles) on 6 October 2021 and 3 March 2022. White lines represent public walking trails **(B)**. A bar plot depicting the relative abundance at the species level for mosquito specimens collected across both trap nights during the study. Specimens from eight distinct species were identified, with the most abundant being *Deinocerites cancer* (34.8% of specimens collected), *Culex nigripalpus* (16.0%), *Aedes taeniorhynchus* (14.4%), and *Anopheles atropos* (12.7%). Colors represent different mosquito taxa **(C)**. The map of Florida was generated from QGIS by Alexandra Bauer. The ORCA map image was adapted from arcgis.com. Mar, March 2022. Oct, October 2021.

Mosquitoes were collected from the Oslo Riverfront Conservation Area (ORCA), 298 acres of public conservation land located north of the Florida Medical Entomology Laboratory (FMEL) in Vero Beach, Florida, USA, which contains hiking trails and an impoundment area managed by the local mosquito control district (**Figure 1A**). Within ORCA, there are several forest types that harbor different flora and fauna, including a large area of mangrove wetlands that abuts the Indian River Lagoon (**Figure 1B**). Previous studies of the area demonstrate high abundance and diversity of mosquitoes in the ORCA mangroves, as well as a high diversity of mosquito-associated bacteria (56), making it an ideal site to investigate the mycobiomes of local mosquito species.

### Mosquito collection and identification

Mosquitoes were collected using CDC mini light traps (John W. Hock Company) baited with dry ice-containing canisters in order to attract host-seeking mosquitoes, as blood feeding has been demonstrated to cause dysbiosis in the mosquito microbiota (17, 61). On each trap night, two light traps were deployed into the ORCA mangrove wetlands and placed approximately 200 m apart. Traps were placed in the field between 15:00 and 18:00 and collected the next day approximately 08:00–10:00. Trapping was conducted during the hot and cooler periods of the year, as previous studies have demonstrated that seasonality and the time of year when samples were collected (collection time) can alter the composition of the mosquito microbiome (13, 16), and we were interested to see if this also occurred for the fungal microbiome. Trap nights were conducted on 6th October 2021, during the end of the Florida summer, and on 3rd March 2022, before temperatures increased again. Data from five environmental parameters [temperature (°C), precipitation (mm), dew point (°C), wind speed (km/h), and sea level pressure (hPa)] were collected three weeks prior to the night of collection from the Weather Underground website (Data from Vero Beach Municipal Weather Station, accessed via Weather Underground).

After collection, the traps were returned to the laboratory, and the collected specimens were knocked down by exposure to freezing temperatures (–20 °C) for 1h. Mosquito specimens were sorted on chilled glass petri dishes, and bycatch, including male mosquitoes, other insects, and debris, was discarded. All retained female mosquito samples were stored in sealed containers and kept at –20 °C prior to identification. Where possible, mosquito samples were identified to the species level (**Supplementary Material S1**) by the first author using a morphological key (58). Samples that were missing important features were identified, if possible, to the genus level. Samples that were in poor condition were classified as “unidentifiable.” Specimens that were missing key features or that were deemed unidentifiable were not considered for fungal profiling.

### Mycobiome sample selection and preparation from mosquito midgut

Mycobial profiling was performed for three mosquito species: *Ae. taeniorhynchus, An. atropos*, and *Cx. nigripalpus* with specimens collected in both trap nights (**Figure 1C**). These three species were selected because they were either highly abundant in our collections, have a known history of medical and/or veterinary importance, or are considered nuisance biters of humans. For each species, we randomly selected 10 good-quality specimens from each trap night for fungal profiling. For *Ae. taeniorhynchus*, only seven specimens were collected during the March trap night; therefore, all of these samples were used for profiling.

Individual female mosquito specimens were dissected, and their midguts were transferred to individual 2.0 ml tubes containing 100 ml of sterile 1× phosphate buffered saline and a sterile glass bead. We decided not to surface sterilize these specimens, as immersion in ethanol could potentially eliminate key members of the mycobiome. To limit cross-contamination, the forceps used to dissect and manipulate mosquitoes were sterilized using a flame and then immersion in 70% ethanol after contact with each individual mosquito. Mosquito midguts were homogenized with a QIAGEN TissueLyser II at 19.5 frequency 1/s for 3 min. DNA from each sample was extracted using a Quick-DNA Miniprep Plus Kit (Zymo, D4068), following the manufacturer’s instructions using the solid tissue protocol. Sample DNA yield and absorbance (A_260/280_ and A_260/230_ ratios) were determined using a Thermo Scientific μDrop Plate (Cat. No. N12391) and Multiskan SkyHigh Microplate Spectrophotometer. Samples with an A_260/280_ ratio of 1.8–2.0 and a concentration of more than 20 ng/μl were considered to be of sufficient quality for sequencing. Two no-sample DNA extractions (kit blanks) were performed in parallel using homogenization buffer, and an aliquot of the final elution buffer was collected as an elution blank. These three samples were also sent for mycobial sequencing and served as negative controls to help identify any microbial contaminants in the data set.

### Fungal profiling and bioinformatic analysis

Fungal profiling was performed for all samples by sequencing the Internal Transcribed Spacer (ITS) region, a highly conserved intergenic sequence between the small (18S/5.8S) and large (5.8S/28S) ribosomal subunits, which is commonly used to metabarcode the DNA of fungi. DNA samples were shipped to MR DNA (Shallowater, TX, USA) to perform PCR amplification for the ITS region using ITS1-F/ITS-2R primers (62). Their services included performing quality assurance, the construction of libraries, conducting 2× 300 bp Illumina MiSeq barcoded amplicon sequencing (63), and using a custom analysis pipeline (64) to generate zero-radius operational taxonomic unit (zOTU) calls for all unique denoised sequences (65). All zOTUs were classified taxonomically against a curated database containing fungal ITS sequences sourced from NCBI. The returned data were subsequently used to analyze and compare the mycobial diversity of mosquito specimens across species and collection times. Raw sequencing data have been deposited in the NCBI Sequence Read Archive (BioProject accession number: PRJNA1274493).

### Mycobial diversity analyses

Sequencing data was analyzed at the class (**Supplementary Material S2**), family (**Supplementary Material S3**), and zOTU (**Supplementary Material S4**) taxonomic levels, ensuring that only fungi were included. Class and family level data were used to generate the mycobiome profile and abundance plots for each individual mosquito specimen and identify highly abundant and prevalent fungal taxa. Read count data at the zOTU level were used to generate multiple alpha and beta diversity metrics and to analyze the influence of the two key test variables, mosquito species and collection time, on mosquito fungal diversity. An abundance threshold of 0.1% of all reads in any sample was applied, and zOTUs below this threshold were deemed to be either contaminants or unlikely to be biologically important and were removed from subsequent analyses (56). For each sample, counts of zOTUs at greater than 0.1% of all reads were generated as a metric of fungal richness. Further diversity analyses were performed using the R package vegan (v. 2.6-4). Three measurements of alpha diversity, the Shannon and Simpson (1 minus the sum of the squared proportional abundance of species) indices, and evenness were calculated using the *diversity()* function. The Chao1 species estimator was performed using the *estimateR()* function. The relative abundance of reads of zOTUs from a key fungal species, *Rhodotorula lamellibrachiae*, was compared between the three mosquito species and across collection times using Kruskal–Wallis analysis of variance (ANOVA), with Dunn’s tests performed as a multiple test correction. The normality of data produced for these indices was assessed by using the Shapiro–Wilk test, and was compared between treatments using Kruskal–Wallis ANOVA, both in GraphPad Prism 10 version 10.0.2. Non-metric multidimensional scaling (NMDS) was used as a metric of beta diversity via the *metaMDS()* function, after generation of a Bray-Curtis distance matrix using the *vegdist()* function. PERMANOVA was used to assess the impact of collection time, mosquito species, and the interaction between those variables on zOTU count data using the *adonis2()* function.

Heat maps depicting the prevalence of key fungal taxa across mosquito species and time of collection were produced using GraphPad Prism. The data were split into six separate treatments based on mosquito species and time of collection. The prevalence of each zOTU in each of those treatments was calculated, and zOTUs with a prevalence of at least 20% in any one treatment were included in the heat map. Rarefaction analyses were performed using the R package iNEXT (v3.0.0) for each mosquito species at each collection time using the *ChaoRichness()* function. Due to differences in read counts, which limited interpretation of the resulting graph, the rarefaction analysis for *An. atropos* was performed independently of the analysis for *Ae. taeniorhynchus* and *Cx. nigripalpus*.

Network analysis was performed to observe relationships between zOTUs due to time of collection and host mosquito species. Count data for each mosquito species (across collection times) and each collection time (across mosquito species) were used to generate matrices of Pearson correlations. For each analysis, any strong correlations greater than 0.5 or less than –0.5 were used to build the network. All statistical analyses were performed using GraphPad Prism version 10.0.2 (171) or R Studio (2023.06.2 + 561). Figures were produced using R, GraphPad Prism, and/or Microsoft PowerPoint. Additionally, Microsoft PowerPoint was used for preparation and editing of multi-panel figures. Rarefaction curves were generated in R, and then low dpi axes text from the R output was removed using GIMP v2.10.24 and replaced in Microsoft PowerPoint. All R scripts are provided in **Supplementary Material S5**. Data generated from analyses and used to prepare figures are provided in **Supplementary Material S6**.

## Results

### Mosquito collection

A total of 2,884 mosquito specimens were collected across the two trap nights, from which 2,827 (98.02%) were identified to species, 32 damaged specimens (1.11%) were identified to genus level, and 25 (0.87%) were unidentifiable (**Figure 1C**). From the October trap night, 2,531 (87.76%) mosquito specimens were collected, from which 408 (16.12%) were *Ae. taeniorhynchus*, 210 (8.29%) were *An. atropos*, and 381 (15.05%) were *Cx. nigripalpus*. During the March collection, a total of 353 mosquito specimens were collected, from which 7 (22.38%) were *Ae. taeniorhynchus*, 156 (40.94%) were *An. atropos*, and 79 (22.38%) were *Cx. nigripalpus.* An average of 6.75 mosquito species were collected per trap night. A significant difference in the mosquito specimens collected between trap nights was observed (Chi-square test: *X^2^* = 49.71, *P* < 0.0001).

### Composition of the mosquito mycobiota

**Figure 2:**
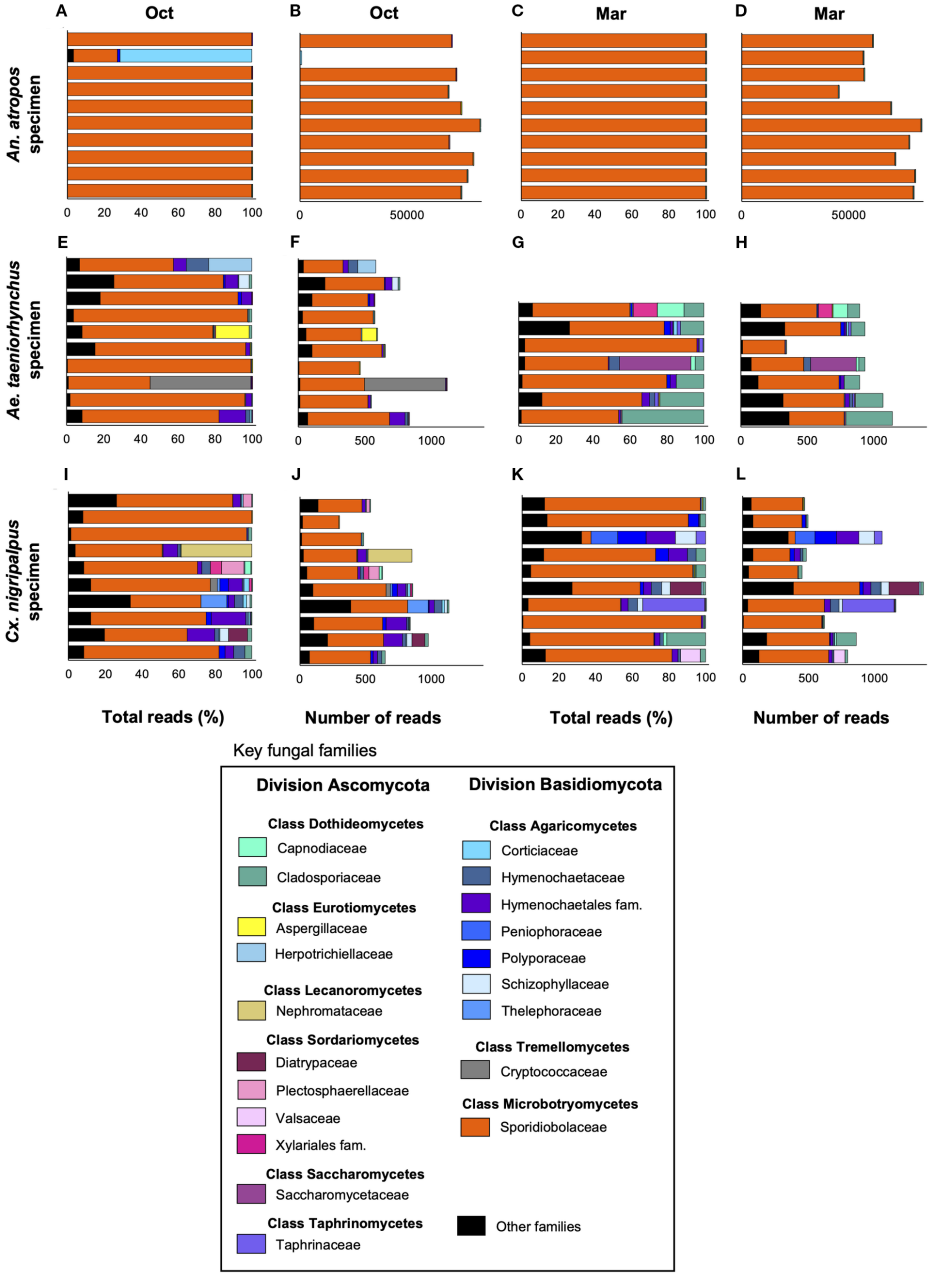
Abundance of key fungal taxa across time and host mosquito species. Bar plots depicting the relative abundance of key fungal families belonging to one of two divisions (Ascomycota and Basidiomycota) as a percentage of total reads **(A, C, E, G, I, K)** or total number of reads **(B, D, F, H, J, L)** as determined via ITS1–2 profiling, for *An. atropos* **(A–D)** *Ae. taeniorhynchus* **(E–H)** or *Cx. nigripalpus* **(I–L)** mosquitoes collected in either October 2021 (Oct), or March 2022 (Mar). Fungi from family Sporidiobolaceae were highly abundant and prevalent across all treatments, but were particularly abundant in *An. atropos* where, on average, they accounted for more than 90% of all reads. Different colors represent different fungal families. Reads from less abundant families were grouped into the Other Families category (black).

Our ITS sequencing profiling generated 2.81 million reads, of which 1,387 could not be mapped. A total of 1.39 million reads were of fungal origin, with an average of 24,411 reads per mosquito sample (median = 873 reads). The average fungal reads by mosquito species were 786 for *Ae. taeniorhynchus*, 68,168 for *An. atropos*, and 735 for *Cx. nigripalpus.* Using both percentage of total reads and total fungal read counts, the mycobiome profiles were generated for individual mosquito specimens, highlighting key fungal taxa representing the two major fungal divisions, Ascomycota and Basidiomycota (**Figure 2**). The three most abundant classes across all species and both collection times were Microbotryomycetes (average of 99.1% of reads per sample), Agaricomycetes (0.485%), and Dothideomycetes (0.147%). At the zOTU level, five zOTUs matched to *Rhodotorula lamellibrachiae* (Class Microbotryomycetes, Family Sporidiobolaceae), with one of these (zOTU1) being highly abundant in every mosquito specimen. The other four *R. lamellibrachiae*: zOTUs were detected in greater than 95% of mosquito specimens. Other zOTUs with high prevalence included three matching to *Cladosporium cladosporioides*: zOTU44 (present in 61.4% of all specimens), zOTU27 (52.6%), and zOTU105 (40.4%); two matching to *Ganoderma orbiforme*: zOTU59 (42.1%) and zOTU83 (21.1%); and two matching to *Coriolopsis caperata*: zOTU54 (29.8%) and zOTU90 (17.5%) were among the taxa with high prevalence.

### Mycobial diversity

From a total of 332 zOTUs, 201 zOTUs were included in the dataset used to perform mycobial diversity analyses. Non-fungal taxa (114 from Phylum Arthropoda, 10 from Clade Streptophyta, 1 from Division Rhodophyta, and 1 from Phylum Gyrista) were excluded from the analysis. Four fungal taxa (*Malassezia globosa, Alternaria alternata, Talaromyces radicus*, and *Lalaria inositophila*) and one “no-hit” sample that appeared in the elution blank controls were considered to be contaminants and excluded from subsequent analyses.

**Figure 3:**
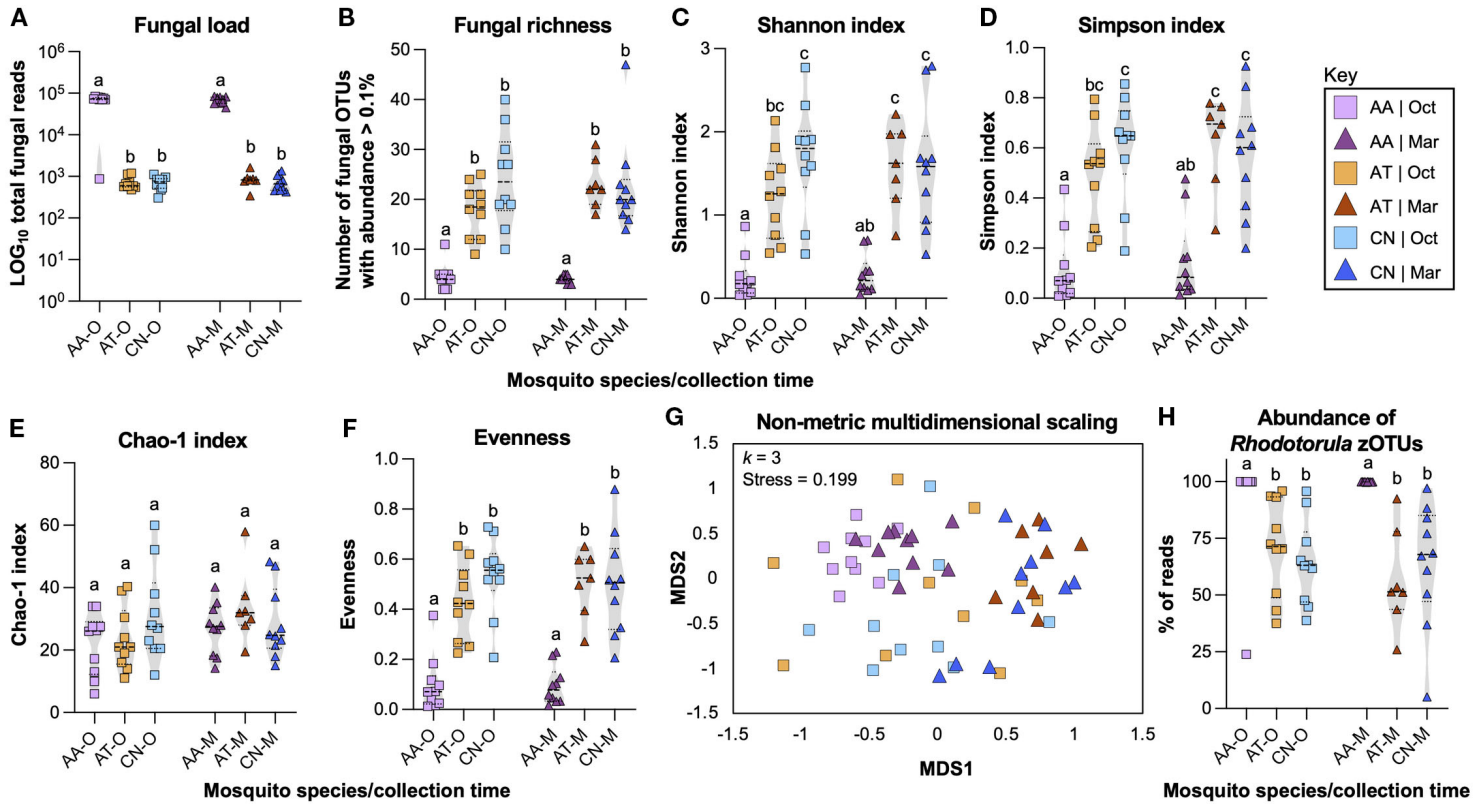
Diversity analyses highlight the influence of mosquito species on the mosquito mycobiome. Fungal ITS1–2 profiling data were used to generate violin dot plots of fungal load (total fungal reads), and five different alpha diversity metrics, while non-metric multidimensional scaling was used as a measurement of beta diversity. All analyses were used to compare the impact of mosquito species and collection time on the mosquito mycobiome. Fungal load, measured as LOG_10_ total fungal reads per mosquito specimen for *An. atropos* (AA, purple), *Ae. taeniorhynchus* (AT, orange), and *Cx. nigripalpus* (CN, blue) collected in October 2021 (O, squares) or March 2022 (M, triangles). Fungal load in *An. atropos* was significantly higher for both time points **(A)**. Violin plot of fungal richness, measured as the number of zOTUs at greater than 0.1% abundance per specimen, revealing significantly lower richness for *An. atropos* mosquitoes at both time points **(B)**. Violin dot plots of Shannon–Weiner **(C)** and Simpson **(D)** diversity indices, depicting lower fungal diversity for *An. atropos* mosquitoes. Violin dot plot of the Chao-1 diversity index highlighting no significant differences between treatments **(E)**. Violin dot plot highlighting lower evenness in the *An. atropos* mycobiome **(F)**. Nonmetric Multidimensional Scaling ordination plot of Bray-Curtis dissimilarity between treatments. Each point represents the mycobiome of a single mosquito specimen. Clustering effects due to species (color) and time of collection (shape) were observed **(G)**. Relative abundance of reads from *R. lamellibrachiae* zOTUs (1, 2, 3, 6, 7, 316, and 332) expressed as a percentage of total reads. Every mosquito specimen in the dataset had reads from one or more of these seven zOTUs. As determined by Kruskal–Wallis ANOVA, *R. lamellibrachiae* abundance levels differed between mosquito species but not by collection time, with significantly higher levels present in *An. atropos* specimens **(H)**. Letters above datasets indicate significant differences between treatment groups as determined via Kruskal–Wallis ANOVA with multiple test corrections. Dashed lines in panels A-G depict treatment medians and interquartile ranges. Dark- and light-colored shapes provide an additional means of differentiation between the October and March collections.

Total fungal reads per specimen was used as a proxy for fungal load, with this parameter significantly higher in *An. atropos* specimens than in the other two species (**Figure 3A**, Kruskal–Wallis ANOVA; *H* = 36.98, *P* < 0.0001), with an excess of reads from *R. lamellibrachiae* zOTU1 is primarily responsible for this difference. The total number of unique fungi per mosquito specimen was higher for *Ae. taeniorhynchus* and *Cx. nigripalpus* than in *An. atropos* specimens (**Figure 3B**, Kruskal–Wallis ANOVA; *H* = 39.74, *P* < 0.0001). Three measurements of alpha diversity were performed: the Shannon–Weiner index, Simpson’s diversity index, and the Chao1 index, as well as the test for evenness. For both the Shannon (**Figure 3C**, Kruskal–Wallis ANOVA; *H* = 36.62, *P* < 0.0001) and the Simpson indices (**Figure 3D**, Kruskal–Wallis ANOVA; *H* = 33.08, *P* < 0.0001), lower diversity was observed for *An. atropos* than the other two mosquito species, and no differences were observed due to collection time. No significant differences in the Chao1 were observed due to mosquito species or collection time (**Figure 3E**, Kruskal–Wallis ANOVA; *H* = 5.851, *P* = 0.3210). Evenness, which is higher when fungal zOTUs have similar abundance, was also significantly lower for *An. atropos* mosquitoes at both collection times (**Figure 3F** **, H** = 36.59, *P* < 0.0001).

NMDS analysis (**Figure 3G** **, k** = 3, stress = 0.199) revealed clustering of samples based on mosquito species, with less extensive separation due to collection time. Additionally, *An. atropos* specimens were more separated from the other two species, indicating that their mycobiome was less similar. PERMANOVA analysis revealed that mycobiome profiles were significantly impacted by mosquito species (*R^2^* = 0.4532, *F* = 23.52, *P* = 0.001) and collection time (*R^2^ =* 0.0309, *F* = 3.21, *P* = 0.016) but not by the interaction of those two variables (*R^2^* = 0.02476, *F* = 1.29, *P* = 0.230). Finally, the abundance of the seven zOTUs associated with *R. lamellibrachiae* (zOTUs 1, 2, 3, 6, 7, 316, and 332) varied significantly between treatments (**Figure 3H**, Kruskal–Wallis ANOVA; *H* = 31.91, *P* < 0.0001), with Dunn’s multiple correction tests revealing that this effect was driven by higher abundance associated with *An. atropos* mosquitoes from both collection times.

**Figure 4:**
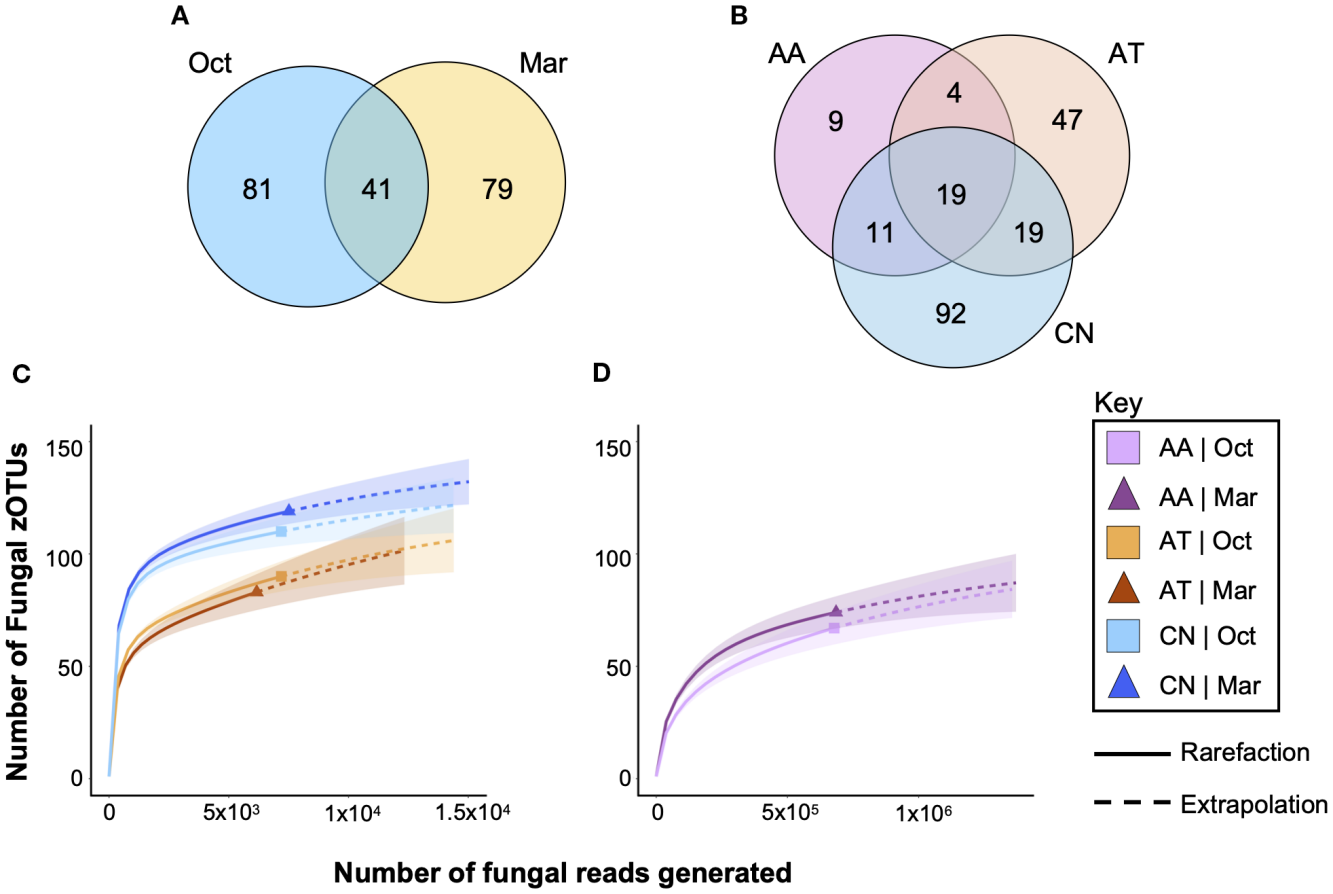
Only a small percentage of fungal zOTUs are shared between mosquito species and across time. Venn diagrams highlight the numbers of fungal zOTUs that are shared between treatments or unique to a treatment for collection time **(A)** and for host mosquito species **(B)**. Only 20.4% of zOTUs were observed in both collection times, while only 9.5% were observed in all three mosquito species, suggesting that mycobiomes in field mosquitoes are highly variable. Rarefaction curves of *Ae. taeniorhynchus* (AT, orange), and *Cx. nigripalpus* (CN, blue) mosquitoes **(C)** and *An. atropos* mosquitoes **(D)**. Analysis suggests that fungal diversity is highest in CN, lower in AT, and lowest in AA mosquitoes, and that differences due to time of collection were most apparent in CN mosquitoes. The graphs show that a small proportion of fungi in these mosquito populations went undetected in our study, approximately 10–20 zOTUs per species. Data were separated into two graphs due to differences in x-axis scale, with far more reads from *An. atropos* necessary to accurately assess fungal diversity due to saturation with *R. lamellibrachiae*. Solid lines – observed data. Dashed lines – extrapolation.

Venn diagrams were constructed to identify zOTUs that were shared across collection time (**Figure 4A**) and across mosquito species (**Figure 4B**). A total of 41/201 zOTUs (20.4%) were found at both collection times, indicating that significant changes to the fungi that associate with mosquitoes occurred between the two collection times. A total of 19/201 (9.5%) zOTUs found in all three species, suggesting that few fungi were common across all species. Interestingly, a total of 34/43 (79.1%) of all zOTUs found in *An. atropos* specimens were also found in at least one of the other two species, indicating that that species had few unique fungi. In contrast, 52.8% of fungal zOTUs found in *Ae. taeniorhynchus* and 65.2% of those found in *Cx. nigripalpus* were unique to those species.

Rarefaction analysis of fungal richness for each treatment (mosquito species × time) revealed greater fungal richness for *Cx. nigripalpus* than for *Ae. taeniorhynchus* (**Figure 4C**). Higher richness was observed for *Cx. nigripalpus* collected in March compared to October, while effects of time were negligible for *Ae. taeniorhynchus*. For both species, extrapolation predicted the presence of an additional 10–20 fungi not detected in our data set. For *An. atropos* (**Figure 4D**), richness was lower than the other two species, but additional fungi were harder to detect due to saturation with *R. lamellibrachiae* reads. Slightly higher richness from the March collection was observed for that species too.

### Fungal community structure

**Figure 5:**
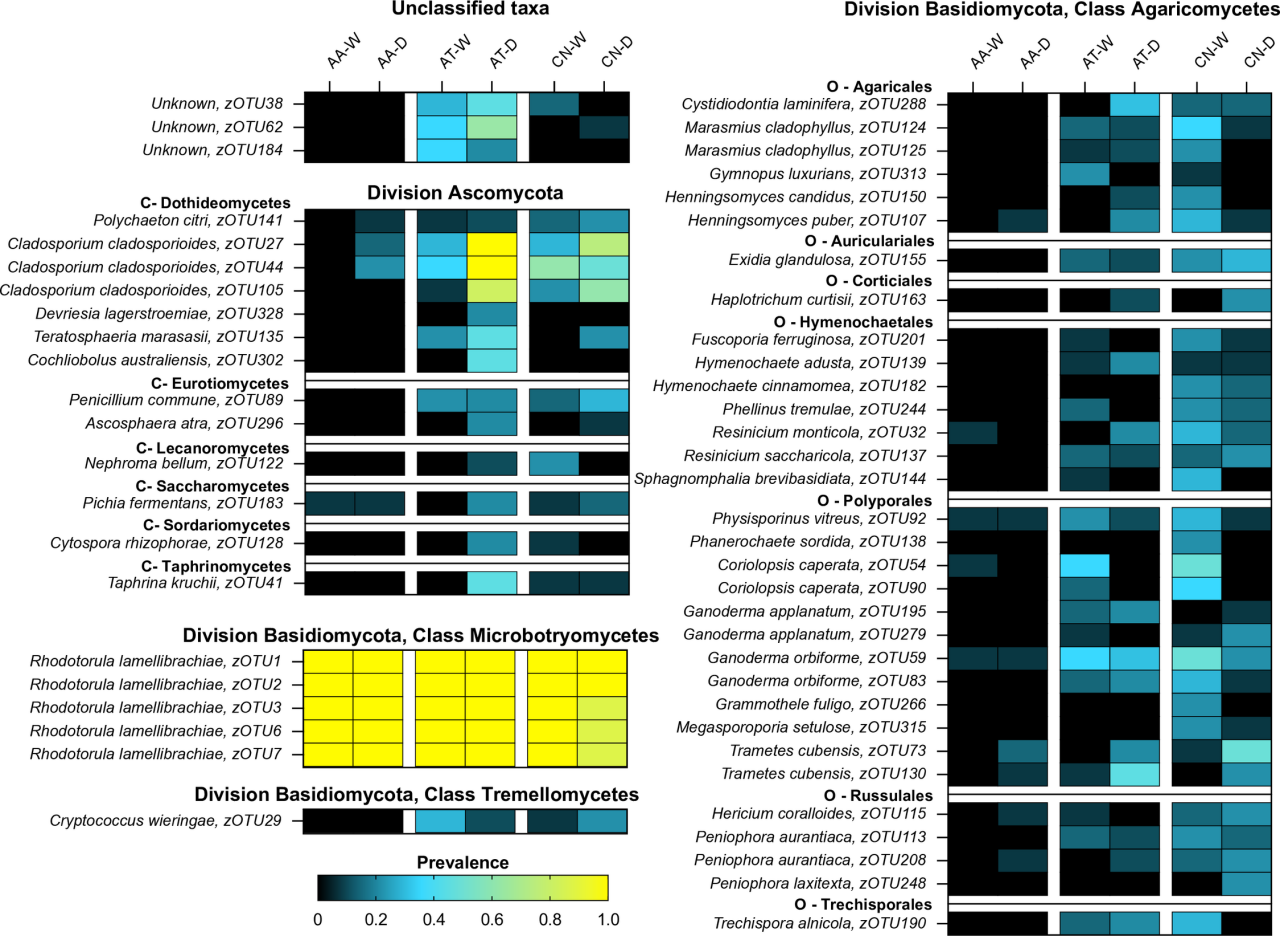
The prevalence of key fungal zOTUs varies between mosquito species and due to time of collection (O – October. M – March). Heatmap depicting changes in prevalence for 54 fungal zOTUs (rows) across treatments (mosquito species × collection time, columns). The zOTUs displayed on the heatmap are those with higher average abundance for each species. The heatmap is split taxonomically between divisions Ascomycota and Basidiomycota, with the latter further sub-divided into three classes (Agaricomycetes, Microbotryomycetes, and Tremellomycetes). Within these groupings, taxa are further subdivided by class, order, family, and then genus. Key zOTUs in Division Ascomycota are further divided by class (C), and for class Agaricomycetes are sub-divided by order (O). Dark colors (black-blue) indicate lower prevalence whereas light colors (green-yellow) indicate higher prevalence. The heatmap reveals that few fungal zOTUs in *An. atropos* have high prevalence, with the exception of five *R. lamellibrachiae* zOTUs. Both *Ae. taeniorhynchus* and *Cx. nigripalpus* have more diverse mycobiomes, with more moderate prevalence zOTUs, but few had high prevalence across both collection times, indicating that fungal communities in these species might assemble randomly, or have a high degree of functional redundancy.

A heat map of 54 of the more abundant fungal zOTUs (**Figure 5**) demonstrates broad differences in the composition of mosquito mycobiomes between species and over time. The most constant feature of the dataset was the high prevalence of *R. lamellibrachiae* zOTUs across all six treatments, highlighting the ubiquity of those yeasts. Aside from those taxa, there were no other fungi of high prevalence found in *An. atropos* specimens at either time point. For *Ae. taeniorhynchus* and *Cx. nigripalpus*, there were many fungi with a prevalence between 0.4 and 0.6, but few with a prevalence higher than 0.6. There were also few fungi with a high prevalence in both collection periods. These findings suggest that there was little consistency and structure to the midgut fungal communities, with the exception of the *R. lamellibrachiae* zOTUs. Exceptions to this include *Cladosporium cladosporioides* zOTUs that had high prevalence in *Ae. taeniorhynchus* and *Cx. nigripalpus* in both seasons, and three unknown zOTUs associated with *Ae. taeniorhynchus* in both time periods. There was also a trend of a greater number of highly prevalent Division Ascomycota zOTUs in *Ae. taeniorhynchus* from the March collection and more high prevalence division Basidiomycota zOTUs in *Cx. nigripalpus* from the October collection.

**Figure 6:**
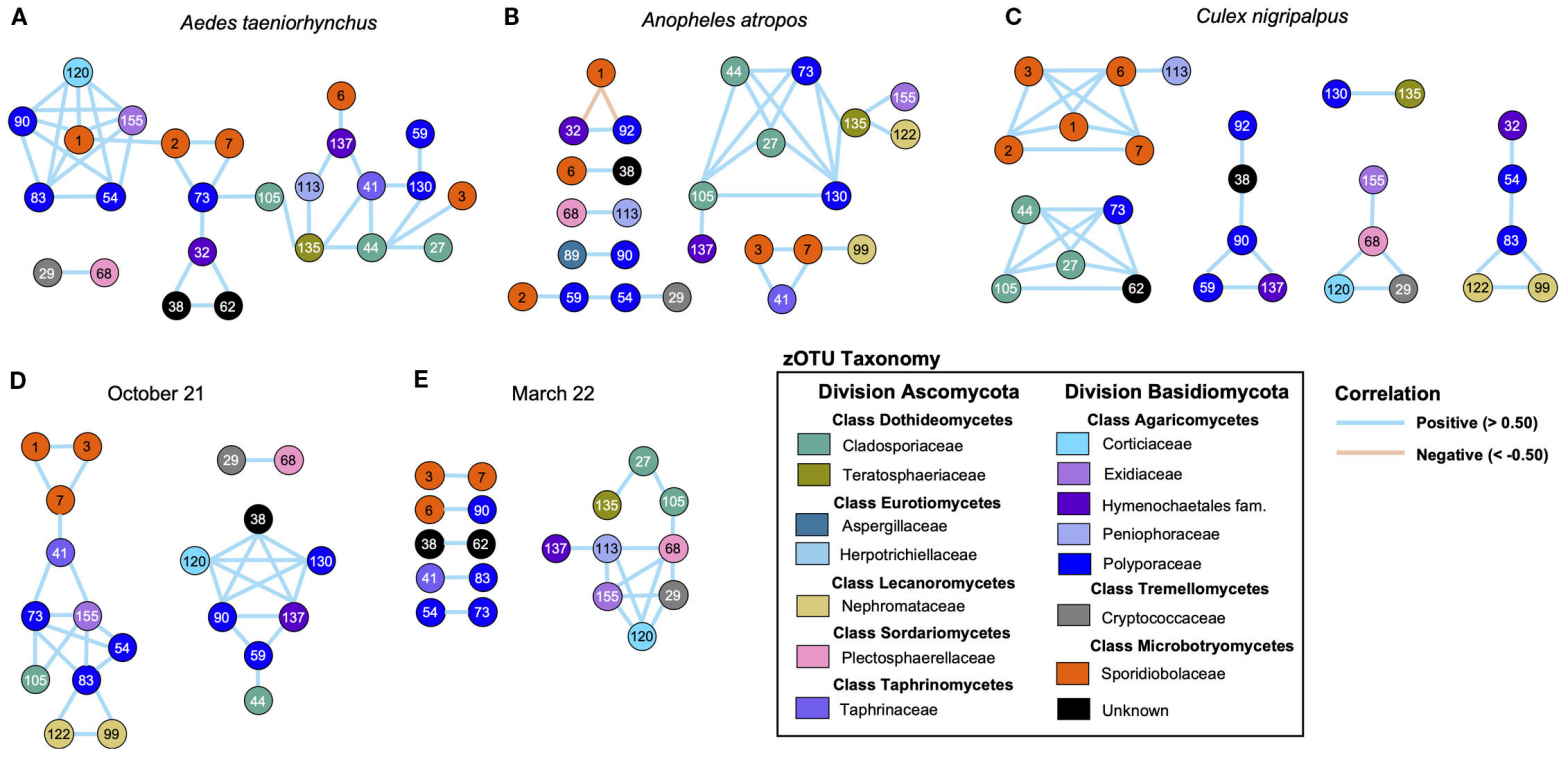
Network analysis reveals differences in fungal community structure between mosquito species, and greater connectivity during the October collection. Network maps based on Pearson correlation matrices of strong positive (> 0.50, blue lines) and negative (< −0.50, orange lines) correlations of fungal read counts across mosquito specimens. Networks were produced for *Ae. taeniorhynchus* **(A)**, *An. atropos* **(B)**, and *Cx. nigripalpus* **(C)** mosquitoes for both collection points, and for the October **(D)** and March **(E)** collection points, encompassing specimens from all mosquito species. Larger clusters and a greater number of connected zOTUs indicate a more structured fungal community, while small clusters with fewer connections indicate a more fragmented community. Based on these criteria, *Ae. taeniorhynchus* mosquitoes had the most structured mycobiome of the three species, and *An. atropos* the least, while mycobiomes from mosquitoes collected in March 2022 were more fragmented than those from October 2021. All correlations in the dataset were positive, except for two observed in *An. atropos*, potentially suggesting that there was limited competition or co-exclusion between fungal taxa. Numbers inside circles are the zOTU number (see **Supplementary Materials** for full list). Circle colors reflect taxonomy, with the same color indicating zOTUs belonging to the same fungal family. Lines between circles indicates a strong correlation between read counts for the two zOTUs.

Pearson correlation matrices produced from fungal read counts were used to identify strong positive (>0.5) or negative (<−0.5) correlations between zOTUs for each mosquito species and for the two collection times. The vast majority of these correlations were positive, suggesting that mosquito-associated fungi in our dataset were unlikely to be competing to fill a niche. *Aedes taeniorhynchus* (**Figure 6A**) had a highly ordered community structure, with 25 zOTUs all positively correlated, and 23 of those found in a single cluster. For *An. atropos* (**Figure 6B**), there were 26 correlated zOTUs, but they were split across seven clusters, and the largest of these contained only nine zOTUs. In this species there was a negative association between *R. lamellibrachiae* zOTU1 and two other zOTUs, the only strong negative association observed in our data. For *Cx. nigripalpus* (**Figure 6C**), 27 positively correlated zOTUs spanned six clusters, with 16 interactions between zOTUs from the same classes. Only two interactions were observed for all three mosquito species: zOTUs 27 and 44 (both *Cladosporium cladosporioides*), and zOTUs 73 (*Trametes cubensis*) and 105 (*Cladosporium cladosporioides*). A comparison of fungal networks for all mosquitoes collected in October 2021 (**Figure 6D**) and March 2022 (**Figure 6E**) revealed a more fragmented network from the March timepoint. Only three connections were shared across the two timepoints: zOTUs 3 and 7 (both *R. lamellibrachiae*), 73 (*Trametes cubensis*), and 54 (*Coriolopsis caperata*), both from the family Polyporaceae, and finally zOTUs 29 (*Cryptococcus wieringae*) and 68 (*Verticillium alfalfae*) from different fungal divisions.

## Discussion

Although there are a large number of studies that highlight the importance of the bacterial microbiota to mosquito biology and vector competence, few have characterized the fungal symbionts, the mycobiota, of mosquitoes, which may have unexplored immunological impacts. Our study describes the mycobiota of adults from three mosquito species, *Ae. taeniorhynchus*, *An. atropos*, and *Cx. nigripalpus*, collected from a mangrove swamp in a central Florida conservation area at two different timepoints. We find that the midguts of these three species harbor many different fungi, but most are of low abundance and prevalence. We also observed that fungal diversity was more strongly impacted by mosquito species than by collection time, and that there was low interconnectivity between taxa, with these factors being suggestive of minimal community structure. However, all specimens from each of the three mosquito species had high levels of the yeast *R. lamellibrachiae*, a widespread environmental microorganism, which is potentially involved in nitrogen fixation (66). These findings contrast with our previous study of the bacterial microbiome of mosquitoes from the same area, which highlighted a greater influence of collection time and higher variation between specimens, with no single dominant microorganism (56). This difference suggests that the nature of host-microbe-environment interactions might differ for mosquito-associated bacteria and fungi.

### The relative importance of collection time and mosquito species on microbial diversity

Analysis of fungal diversity repeatedly revealed a stronger impact of host species than collection time on the mosquito mycobiome. Significant host species effects on fungal richness, Shannon diversity, and Simpson diversity were observed, with the differences likely explained by lower fungal diversity in *An. atropos* mosquitoes. Ordination analysis revealed strong clustering of *An. atropos*, and some loose clustering by species and time for the other two mosquito species. These differences were likely caused by the high abundance of *R. lamellibrachiae* reads in *An. atropos* specimens, with 1–2 orders of magnitude more reads observed for that species. As a result, the total number of fungal zOTUs detected across all *An. atropos* specimens were less than half of the number observed in each of the other two mosquito species. This oversaturation could foreseeably have limited the ability to detect reads from other fungi that were present in the midgut. Alternatively, a heavy infection of *R. lamellibrachiae* may have limited the ability of other fungi to inhabit the midgut of that species. Interestingly, we found that the majority of fungal zOTUs detected in *Ae. taeniorhynchus* and *Cx. nigripalpus* were unique to those species, and that less than 10% of all fungal zOTUs in the dataset were found in all three species. Collectively, these observations suggest that there is a strong impact of host species on the fungi that inhabit a mosquito midgut.

Mosquitoes acquire the majority of their bacterial microbiome during larval development (53), while the pathways for the acquisition of fungi may be more variable. Some fungi are acquired during juvenile development and others by adults through behaviors such as nectar feeding and resting. Modes of fungal infection include both cuticular penetration and ingestion. For example, *Zancudomyces culisetae*, a fungal mutualist found in mosquito guts, colonization occurs in larvae and influences both larval size and development success (67, 68). Yeasts, including those from the genus *Candida*, which are common to the *Aedes albopictus* mycobiome, are found in the nectar of flowering plants and might be imbibed during nectar foraging (54). Similarly, *Rhodotorula mucilaginosa*, which has been found in adult *Culex quinquefasciatus*, can be found in human drinking water (69, 70). Our dataset included fungal zOTUs associated with soil, water, and plants, all of which were found in adult mosquito midguts. While the exact methods of colonization have not been evaluated in this study, our data support the hypothesis that fungi might come to associate with mosquitoes from a variety of environmental sources.

The results of our PERMANOVA revealed that mosquito species explained 45.3% of the variation in our data, while time of collection accounted for only 3.1%. Our previous study of the bacterial microbiome of mosquitoes collected from the same area revealed that time of collection explained 6.5% of the total variation, while mosquito species explained only 4.4% (56). That result echoed the findings of other studies indicating that mosquito species is not the most important predictor of bacterial microbiome composition (51, 61). Our data suggest this may not be the case for fungi. A study of the mycobiota of *Aedes albopictus* larvae in Kansas, United States, revealed high diversity and a strong influence of the local environment on fungal community structure (52), but did not examine differences between host species. The data from our two studies comprise a fairly robust sample size (57 fungal mycobiomes and 94 bacterial microbiomes) but represent mosquitoes from only a single region in Florida. Nevertheless, our data potentially highlight differences in the ability of fungi and bacteria to find suitable niches in mosquito biology, with fungi potentially being subject to greater species specificity and bacteria potentially showing greater ubiquity across mosquito species. To reinforce this point, the average richness (number of zOTUs with abundance greater than 0.1% of reads) across these two studies was 56.3 for bacteria but only 15.6 for fungi, indicating that the average mosquito harbored about 3.6 times fewer unique fungi than unique bacteria per mosquito. Given the dearth of studies of the mosquito mycobiome, it is unclear if this differential is widespread or what the biological implications might be; however, it might suggest that the niches for fungi in mosquito biology are more narrow than those for bacteria.

### Impact of *Rhodotorula lamellibrachiae* on mosquito biology

The most ubiquitous microorganism that we detected in our data was *R. lamellibrachiae*, which was found in every mosquito specimen that we sequenced but appeared to have a strong ability to proliferate in the midguts of *An. atropos*. This Basidiomycota fungus is a common environmental microorganism, has distinct orange-pink pigmentation when cultured, and is a carbon and nitrogen scavenger capable of feeding on a broad range of substrates (66). Some *Rhodotorula* species are known to be opportunistic pathogens affecting immunocompromised people (71). Others have been explored for potential use in bioremediation due to their capacity to degrade petroleum and survive high levels of radiation (72–74). *Rhodotorula* species have been explored as feedstock for oleochemical production (75). They can also be found in other insect species, where they have been demonstrated to impact host fitness. In *Drosophila suzukii*, the presence of *Rhodotorula mucilaginosa* increases larval development time, while in the bumble bee *Bombus terrestris*, it increases the number of workers but decreases mating success and body mass (76, 77).

Yeasts, including *Rhodotorula* spp., have been identified in several field-collected mosquito species (78, 79), although their role in mosquito biology is still being elucidated. A recent study suggests that *R. mucilaginosa* on the cuticle of *Ae. albopictus* can directly metabolize deltamethrin, and its inoculation onto the cuticle can enhance mosquito survival after pyrethroid exposure (80). The same yeast species has been observed to decrease juvenile development time in *Ae. albopictus*, which was hypothesized to be due to the provisioning of the B vitamin riboflavin to the mosquito by the yeast (81). B vitamin provisioning is a characteristic of successful bacterial symbionts in mosquitoes, is vital for juvenile mosquito development, and can produce many positive fitness outcomes for the host and impact vector competence (23, 82). Nitrogen scavenging is another common biological strategy for bacteria in insects (83), and likely mosquitoes, given that nitrogen derived from proteins is abundant in larvae and in blood-fed adult females. Nitrogen scavenging by the bacterium *Wolbachia* has been implicated in reduced fecundity and fertility in *Ae. aegypti* (84). Nitrogen is also important to immune signaling pathways, including those that involve Toll-like receptors, with reactive nitrogen species forming a core part of the mosquito antiviral immune response (85). To date, metabolic and immunological interactions between *R. lamellibrachiae* and mosquitoes and any biological consequences of those interactions have not been characterized, but given its abundance in this dataset, it would likely merit future investigation. Recent studies now group *R. lamellibrachiae* within the genus *Sakaguchia* (86), a less well-explored genus of fungi. Accordingly, it is currently unclear whether this species possesses the same capacity for nitrogen scavenging and lipid and B vitamin provisioning as other members of its former genus.

### Fungal community structure and putative role of other fungal community members

Other key fungi in our dataset belong to families Polyporaceae and Cladosporiaceae. The Polyporaceae (Division Basidiomycota, Class Agaricomycetes), are a well-studied family known for their role in the decomposition and nutrient recycling of plant matter in forested areas (87). Four zOTUs from this clade, *Trametes cubensis* (zOTU73/zOTU130) and *Ganoderma orbiforme* (zOTU59/zOTU83), appear repeatedly in our network analysis due to their high prevalence and abundance. Interestingly, *T. cubensis* is an edible fungus known for its ability to decompose wood matter, and because it can induce anti-inflammatory responses (88). It also produces compounds that can inhibit superoxide anion generation and arrest cancer cell growth (89, 90). However, it is unclear whether this fungus impacts superoxide production and cell growth in mosquitoes. *Ganoderma orbiforme* is also involved in plant decomposition and nutrient recycling, is a plant pathogen (91), and may feed on plant matter undergoing digestion in the mosquito gut. *Ganoderma* spp. are abundant in the *Ae. albopictus* gut (52). Three zOTUs common amongst our specimens (zOTU27, zOTU44, and zOTU105) were from the family Cladosporiaceae (Division Ascomycota, Class Dothideomycetes). Fungi from this family appear to be common amongst mosquito species, having been detected in *Ae. albopictus, Cx. quinquefasciatus*, and *Culex pipiens* (49, 92). The three zOTUs in our dataset all belonged to the species *Cladosporium cladosporioides*, a common mold, which is known for its impact on human health (93) and for its role as a plant pathogen (94). It has been studied as an entomopathogen against hemipteran pests of agricultural importance (95). These three zOTUs all have high prevalence in *Ae. taeniorhynchus* and, to a lesser extent, in *Cx. nigripalpus*, but it is unclear if they can affect the survival of these species after ingestion.

Our assessment of fungal prevalence and fungal networks across mosquito species and collection times reveals little by way of consistent fungal community structure. With the exception of those mentioned above, few fungal zOTUs had high prevalence or abundance, and as evidenced by the heatmap (**Figure 5**), patterns of prevalence varied greatly between collection times, suggesting that there was low consistency in community structure over time. Additionally, the vast majority of strong interactions from fungal taxa were positive, with the exception of two interactions in *An. atropos* between *R. lamellibrachiae* (zOTU1) and *Physisporinus vitreus* (zOTU92, family Polyporaceae) and *Resinicium monticola* (zOTU32, order Hymenochaetales), both known to grow on wood (96, 97). This disparity suggests that the majority of fungi were either not consistently found in the same mosquito, or perhaps that there is little competition between fungi for resources within a mosquito midgut. Network analysis also revealed that there were few strongly correlated pairs of zOTUs observed over multiple treatments: only three pairs for both collection times and two pairs for all three mosquito species. These networks were also highly fragmented, connecting a median of only 3.5 zOTUs. Low connectivity and little consistency between treatments suggest that there may not be a broader pattern underlying fungus–fungus interactions in mosquitoes, at least in our data, which stands in contrast to networks of mosquito-associated bacteria, which display greater interconnectivity (98).

Further study is needed to determine whether such low fungal community structure is common amongst field mosquito populations and to more rigorously elucidate the metabolic and immunological niches held by fungi within mosquitoes. A study of the *Ae. albopictus* mycobiome suggests that the gut mycobiome has a distinct community structure compared to other tissues and is more reflective of the environmental mycobiome (52), with these findings being reflective of our own. Interestingly, network analyses of environment-associated bacterial microbiomes, including those from soil specimens, are typically highly nodular and demonstrate high degrees of interconnectivity compared to those based on fungal communities (99, 100). However, the presence of key fungi shapes the immunological state of a mosquito and mosquito tissues, with potential consequences for other microorganisms. For example, entomopathogenic fungi from the genera *Beauveria* and *Isaria* prompt a reduction in reactive oxygen species levels, which facilitates proliferation of other microorganisms in the *Ae. aegypti* midgut (38), suggesting that currently undescribed interactions between bacteria and fungi might play critical roles in the composition and potentially the functionality of insect microbial communities. Low community structure in such networks may be indicative of a high degree of functional redundancy, where functional roles can be filled by many different fungi, or it may be due to high variation between specimens, with fungal communities assembling in a stochastic rather than deterministic manner, depending on local environmental factors. Accordingly, there is merit to continuing the exploration of fungal diversity in mosquitoes, including those from distinct environments, mosquito tissues, and male mosquitoes.

### Study limitations

Several factors may have influenced the findings of our study. Uncontrolled environmental parameters, including temperature, rainfall, and relative humidity, might have contributed to differences in mosquito abundance and microbial diversity between collection times. Lower than anticipated collections of *Ae. taeniorhynchus* during March meant that we could not perform randomized selection of specimens from that collection. Mosquitoes were collected using CDC light traps baited with dry ice to collect host-seeking rather than blood-fed mosquitoes, as blood feeding can cause dysbiosis of the bacterial microbiota (61). No blood-fed midguts were included in the specimens that were sequenced, but we also did not perform ovarian dissections to assess for parity, which would determine if the mosquito had blood-fed previously. There are also potential limitations associated with sequencing and bioinformatics, which can be impacted by the choice of sequencing platform, primers, and database. The Illumina Mi-Seq platform utilizes shorter read lengths and can therefore be impacted by sequence changes and errors more strongly than other platforms. Low sequencing coverage for many zOTUs may have limited our ability to assess the true nature of correlations and interactions between fungal taxa. Alternative primer sets for fungi, including those based on ITS3 and ITS4, may have led to alternative zOTU calls, and the same is true for queries against a different database of sequences.

## Conclusions

Our findings reveal important insights into the nature of fungal communities within the midguts of mosquitoes in nature. These communities are diverse, but less so than mosquito-associated bacteria. They are highly variable between different mosquito species, with only a low percentage of fungal taxa common across all of the species that we examined. Fungal community networks are fragmented and largely comprised of positive co-associations. These findings suggest that fungal communities are less structured than bacterial communities in mosquitoes. Of further interest is the high abundance and prevalence of the yeast *R. lamellibrachiae* in our data. Ubiquity across mosquito specimens might make it an appealing organism for control of mosquito populations through paratransgenesis, while significantly greater abundance in *An. atropos* mosquitoes might point to key immune differences with the other species in our study. The functional roles that *R. lamellibrachiae* and the other fungi identified in our data hold in mosquito biology are still unclear. Many appear to be saprotrophic, which raises the question of whether these organisms are held over from the juvenile stages when mosquitoes more commonly feed on plant matter, or if they were acquired in the adult stages. Understanding how and when mosquitoes acquire fungi, and the roles that fungi play in mosquito biology, will provide greater insight into the potential impact of these microorganisms on the ability of mosquitoes to spread disease.

## Data Availability

The datasets presented in this study can be found in online repositories. The names of the repository/repositories and accession number(s) can be found in the article/**Supplementary Material**.
