# Supplementary material for: Fungal communities in Florida salt marsh mosquito midguts vary between species and over time but have low structure
Source: Front Immunol. 2025 Sep 23;16:1648091. doi: 10.3389/fimmu.2025.1648091 (PMC12500438; doi:10.3389/fimmu.2025.1648091)
Supplement: Supplementary file 5 [file Table5.docx]

**Supplementary File 5 – R Scripts**

*NOTE_1: The following scripts were used to produce alpha and beta diversity analyses as well as rarefaction curves and PERMANOVA.*

*NOTE_2: X02_zOTUs is the name of the zOTU count matrix used in the analyses.*

library(vegan)

library(fossil)

library(iNEXT)

library(ggplot2)

### Calculation of alpha diversity ###

#Shannon Index

shannon<-diversity(X02_zOTUs_Counts,index = "shannon")

shannon

plot(shannon)

#Simpson Index

simpson<-diversity(X02_zOTUs_Counts,index = "simpson")

simpson

plot(simpson)

#Chao's Species Estimator

data_richness <- estimateR(X02_zOTUs_Counts)

data_richness

plot(data_richness)

#Eveness Index

data_eveness <- diversity(X02_zOTUs_Counts) / log(specnumber(X02_zOTUs_Counts))

data_eveness

plot(data_eveness)

### Calculation of beta diversity ###

#NMDS Analysis

NMDS<-metaMDS(X02_zOTUs_Counts,k=2)

NMDS

plot(NMDS)

NMDS2<-metaMDS(X02_zOTUs_Counts,k=3)

NMDS2

plot(NMDS2)

### Permanova

sqrt_matrix<-sqrt(X02_zOTUs_Counts)

braydm<-vegdist(sqrt_matrix,"bray")

hist(braydm)

set.seed(36)

perm.all2<-adonis2(formula = braydm~Time*Species,data=X03_Vars,permutations=999,method="bray")

perm.all2

### Rarefy with iNEXT

ChaoRichness(X04_zOTUs_Counts_RareFac)

df <- data.frame(X04_zOTUs_Counts_RareFac)

df

Chao <- iNEXT(df, q=0, datatype = "abundance")

ggiNEXT(Chao,)
